# Draft genomes of two phylotype I and II *Ralstonia solanacearum* species complex (RSSC) isolates causing bacterial wilt in tomato plants from Costa Rica

**DOI:** 10.1128/mra.01042-23

**Published:** 2024-01-31

**Authors:** Fiorella Galiano-Murillo, Vidal Salas-Lara, Carlos Echandi, Laura Brenes-Guillén, Lorena Uribe-Lorío

**Affiliations:** 1 Centro de Investigación en Biología Celular y Molecular, Universidad de Costa Rica, San José, Costa Rica; 2 Estación Experimental Agrícola Fabio Baudrit Moreno, Universidad de Costa Rica, San José, Costa Rica; 3 Escuela de Agronomía, Universidad de Costa Rica, San José, Costa Rica; Rochester Institute of Technology, Rochester, New York, USA

**Keywords:** genomes, phytopathogens, *Ralstonia*

## Abstract

Bacteria from RSSC hold agricultural significance as they are the causal agents of bacterial wilt. Here, we report the draft genomes of two bacteria extracted from vascular tissues of infected tomato plants. Isolate RALF-MA was classified as *Ralstonia pseudosolanacearum* (phylotype I) and RALSA-MA as *Ralstonia solanacearum* (phylotype II).

## ANNOUNCEMENT

Tomato plays a crucial role in Costa Rican agriculture; however, bacterial wilt caused by the RSSC substantially threatens its production yields (1). Here we report the draft genomes of two isolates within this complex, obtained from tomato plants exhibiting wilting symptoms. RALF-MA was isolated from the Fabio Baudrit Moreno Agricultural Experiment Station, University of Costa Rica (UCR) (Alajuela), and RALSA-MA from a tomato field in Santa Ana (San José) in November 2020.

Both bacteria were isolated from surface-disinfected stem fragments. The fragments were agitated in sterile deionized water at 100 rpm for 30 min at room temperature. Subsequently, the solution was streaked onto nutrient agar (NA). Colonies exhibiting the distinctive morphology of *Ralstonia* were isolated, purified, streaked onto TZC medium, and cultivated for 72 hours at 30°C. According to the Multiplex PCR (2), RALF-MA is classified as phylotype I, while RALSA-MA as phylotype II and based on the species classification method (3), RALF-MA belongs to *R. pseudosolanacearum*, and RALSA-MA to *R. solanacearum* species.

**Fig 1:**
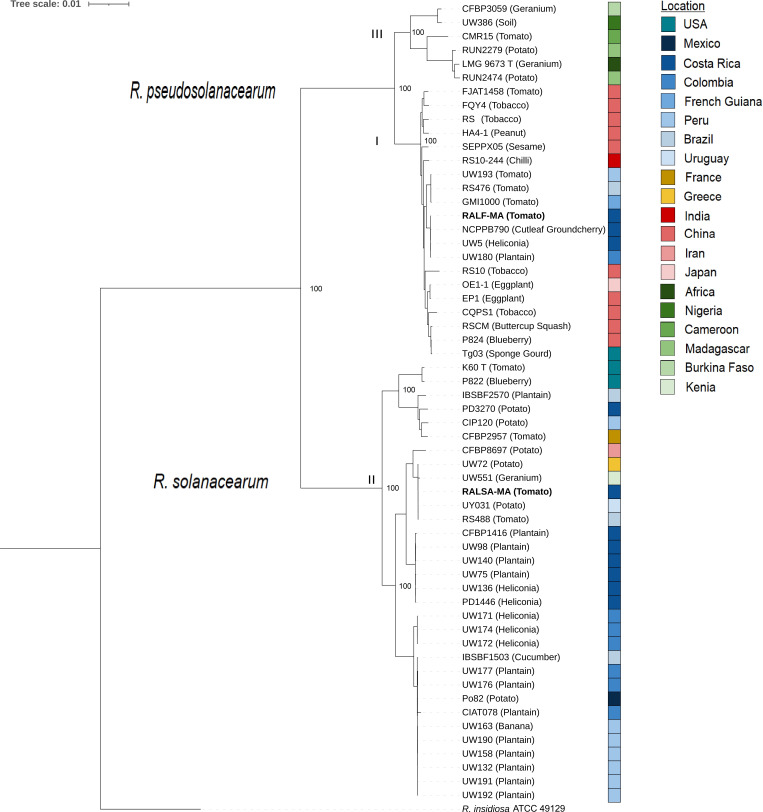
Phylogenetic tree derived from the alignment of protein sequences from 1427 single copy genes. The tree was inferred using the amino acids sequences through Multiple Sequence Alignment (MSA) with the “-M msa” option (9). FastTree was used to conduct maximum likelihood (ML) tree inference (10). Bootstrap values are shown in the tree. Host information is enclosed in parentheses, the color range represents the geographic locations, and I, II, and III indicate phylotypes. *R. insidiosa* was the outgroup.

Genomic DNA was extracted from a 72-hour nutrient agar culture grown at 30°C, using the phenol-chloroform extraction method (4). Sequencing was performed using the NovaSeq 6000 Sequencing System (150 PE) at Novogen Inc. (CA, USA). For all software, unless otherwise specified, default parameters were used. The sequence reads were filtered using Trimmomatic v 0.36 (5) (SLIDINGWINDOW:4:20 and MINLEN of 100 bp), yielding 6,114,942 and 4,366,443, respectively. *De novo* genome assembly was obtained using Unicycler v0.4.9 (6), and the NCBI Prokaryotic Genome Annotation Pipeline (PGAP) was employed to annotate the contigs. To assess the average nucleotide identity (ANI), we employed PyANI (7) for genome comparison, incorporating an additional set of 56 published genome sequences from RSSC. We constructed a core phylogeny using protein sequences from orthologous single-copy genes of RALF-MA and RALSA-MA, in conjunction with the aforementioned 56 genome sequences (Fig. 1). This analysis was conducted using OrthoFinder (8).

RALF-MA draft genome comprised 148 contigs totaling 5,838,634 bp, with an N50 value of 136,125, a G + C content of 66.9%, and a 100× coverage. In comparison, the RALSA-MA assembly had a total length sequence of 5,201,184 bp into 152 contigs, with an N50 value of 108,706, a G + C content of 66.8%, and a 100× coverage.

ANI values revealed that the RALF-MA isolate exhibited a 99.9% identity to UW5 from Costa Rica, UW180 from Colombia, and 99.5% to NCPPB790 (Costa Rica). These strains, isolated between 1955 and 1960 (11) from different hosts, formed a monophyletic clade and displayed closely relatedness to the GMI1000 strain (French Guiana) (Fig. 1).

RALSA-MA exhibited only 96.0% identity with the type strain of *R. solanacearum* but was 99.9% identical with phylotype IIB strain UW551 (Geranium) from Kenya, and UW72 (Potato) from Greece, placing them in a clade with UY031 and RS488 from Uruguay and Brazil, respectively (Fig. 1).

## Data Availability

The genome sequences for RALF-MA and RALSA-MA strains were deposited at DDBJ/ENA/GenBank under the accession numbers JASSVI000000000 and JASSVH000000000, respectively, in the BioProject PRJNA898399. The raw data are available under the SRA accession numbers SRR24727158 and SRR24727157.
